# In Regard to Huang et al

**DOI:** 10.1016/j.adro.2025.101955

**Published:** 2026-03-13

**Authors:** Ilker Sengul, Demet Sengul

**Affiliations:** a Division of Endocrine Surgery, Department of General Surgery, Giresun University Faculty of Medicine, Giresun, Turkey; b Department of Pathology, Giresun University Faculty of Medicine, Giresun, Turkey

We read with great interest the research article “The influence of pelvic bone dose-volume parameters on bone marrow suppression during radiation therapy in patients with stage i to iii rectal cancer based on real-world data,” published in *Advances in Radiation Oncology.*^1^ This study addresses a clinically crucial issue: the hematologic toxicity (HT) experienced by patients undergoing radiation therapy (RT) for rectal cancer. Given the significant role RT plays in treating locally advanced rectal cancer and the potential for HT to disrupt treatment, investigating predictors of this toxicity is valuable.^1^ Huang et al^1^ conducted a retrospective study examining 130 cases with stage I to III rectal cancer who received neoadjuvant RT or curative intent RT. To this end, they evaluated the association between dose-volume parameters across different pelvic bone marrow (PBM) subsites (iliac bone marrow [IBM], lumbosacral bone marrow, and lower PBM) and the incidence of grade ≥ 2 HT. As such, the study reports several significant findings from multivariate analysis. Notably, female sex and higher IBM-Dmean, IBM-V15, and IBM-V40 were identified as independent prognostic factors for grade 2+ leukopenia. Of note, the combination of sex and IBM parameters showed a satisfactory predictive ability for a greater incidence of grade 2+ leukopenia, with an area under the curve of 0.834.^1^ Huang et al^1^ reported that oxaliplatin-containing concurrent chemotherapy regimens were potential factors for increasing the incidence of grade 2+ thrombocytopenia, exhibiting a predictive ability with an area under the curve of 0.678. Herewith, women were also significantly associated with grade 2+ anemia and total grade 2+ HTs. These findings align with the understanding that radiation can damage hematopoietic stem cells in the bone marrow, leading to HT. The identification of specific dose-volume parameters, such as IBM-Dmean, IBM-V15, and IBM-V40, along with patient characteristics, such as sex and chemotherapy regimen, because predictors provides valuable information for clinical consideration. However, it is essential to consider the limitations of this study amiably, which the authors acknowledge. As a retrospective, single-center analysis, the results may be subject to selection bias and other limitations inherent in this type of study design. In addition, the sample size of 130 cases, while providing initial insights, is described as "modest," which necessitates caution when generalizing the findings. The authors appropriately call for external validation of a larger cohort and for prospective randomized controlled trials to confirm their findings, which are notable steps toward strengthening the evidence and establishing reliable dose constraints or predictive models for HT in this patient population. Despite the limitations, this up-to-date study provides valuable real-world data supporting the concept that PBM dose is associated with HTs and highlights the importance of PBM-sparing RT, particularly for vulnerable subgroups such as female patients receiving concurrent oxaliplatin-based regimens. In conclusion, Huang et al's^1^ study contributes to the growing body of evidence linking PBM dosimetry to HT in rectal cancer patients undergoing RT. Although the retrospective and single-center nature warrants further validation, the specific dose-volume parameters and clinical factors identified are essential starting points for developing strategies to mitigate treatment-related toxicity. Future prospective studies building on these findings are essential to refine personalized treatment approaches and supportive care strategies. This issue merits further investigation. We thank Huang et al^1^ for their meritorious study on the division of colorectal diseases^2^ in *Advances in Radiation Oncology*.

## Disclosures

The authors declare that they have no known competing financial interests or personal relationships that could have appeared to influence the work reported in this paper.
